# Interaction of the Onset of Spring and Elevated Atmospheric CO_2_ on Ragweed (*Ambrosia artemisiifolia* L.) Pollen Production

**DOI:** 10.1289/ehp.8549

**Published:** 2006-02-09

**Authors:** Christine A. Rogers, Peter M. Wayne, Eric A. Macklin, Michael L. Muilenberg, Christopher J. Wagner, Paul R. Epstein, Fakhri A. Bazzaz

**Affiliations:** 1 Department of Environmental Health, Harvard School of Public Health, Boston, Massachusetts, USA; 2 New England School of Acupuncture, Watertown, Massachusetts, USA; 3 New England Research Institutes, Watertown, Massachusetts, USA; 4 Center for Health and the Global Environment, Harvard Medical School, Boston, Massachusetts, USA; 5 Organismic and Evolutionary Biology, Harvard University, Cambridge, Massachusetts, USA

**Keywords:** allergenic pollen, *Ambrosia artemisiifolia*, climate change, climate variability, elevated CO_2_, global warming, ragweed, spring-time warming

## Abstract

Increasing atmospheric carbon dioxide is responsible for climate changes that are having widespread effects on biological systems. One of the clearest changes is earlier onset of spring and lengthening of the growing season. We designed the present study to examine the interactive effects of timing of dormancy release of seeds with low and high atmospheric CO_2_ on biomass, reproduction, and phenology in ragweed plants (*Ambrosia artemisiifolia* L.), which produce highly allergenic pollen. We released ragweed seeds from dormancy at three 15-day intervals and grew plants in climate-controlled glasshouses at either ambient or 700-ppm CO_2_ concentrations, placing open-top bags over inflorescences to capture pollen. Measurements of plant height and weight; inflorescence number, weight, and length; and days to anthesis and anthesis date were made on each plant, and whole-plant pollen productivity was estimated from an allometric-based model. Timing and CO_2_ interacted to influence pollen production. At ambient CO_2_ levels, the earlier cohort acquired a greater biomass, a higher average weight per inflorescence, and a larger number of inflorescences; flowered earlier; and had 54.8% greater pollen production than did the latest cohort. At high CO_2_ levels, plants showed greater biomass and reproductive effort compared with those in ambient CO_2_ but only for later cohorts. In the early cohort, pollen production was similar under ambient and high CO_2_, but in the middle and late cohorts, high CO_2_ increased pollen production by 32% and 55%, respectively, compared with ambient CO_2_ levels. Overall, ragweed pollen production can be expected to increase significantly under predicted future climate conditions.

Global climate changes, driven by increased concentrations of greenhouse gases such as carbon dioxide, are having widespread impacts on biotic systems, including both direct and indirect effects on human health (Epstein 1999; Patz et al. 2005). One of the most dramatic effects of climate change seen thus far is on the timing of reproductive processes in plants (Fitter and Fitter 2002), including wind-pollinated types, many of which have highly allergenic pollen (Clot 2003; van Vliet et al. 2002). Hence, predicted increases in CO_2_, coupled with further changes in climate, could have important implications for individuals with allergies and asthma.

Many regions are currently experiencing warming effects associated with global climate change, including longer growing seasons and earlier arrival of spring (Intergovernmental Panel on Climate Change 2001; Karl and Trenberth 2003; Menzel 2000). These changes have already greatly affected plant and animal populations by significantly influencing interannual population dynamics and phenology (Loeuille and Ghil 2004; Root et al. 2003). Analysis of temporal events since the 1950s, across a wide array of plant and animal species, indicates that spring phenology in northern temperate zones is advancing about 5 days each decade (Root et al. 2003). The trend toward earlier spring onset is particularly evident in early spring flowering of wind-pollinated tree species, for which reproductive development and bud burst in spring are highly temperature sensitive (Clot 2003; van Vliet et al. 2002). However, early spring onset may also affect temperature-dependent processes occurring over the entire growing season, not just those in early spring. For example, an early spring could also influence developmental and reproductive processes in later flowering plants.

Although atmospheric CO_2_ has no apparent direct effect on human health, it does have well-known direct effects on plants. Plants grow larger, use water more effectively, and reach maturity faster when grown in elevated CO_2_ (Bazzaz 1990; Drake et al. 1997). In addition, several recent studies suggest that plants can also have an enhanced reproductive effort (Jablonski et al. 2002; LaDeau and Clark 2001; Stiling et al. 2004). These effects are generally thought to be beneficial in agriculture (Southworth et al. 2002); however, some studies suggest this enhanced reproductive effort can also lead to an increase in pollen production (Wayne et al. 2002; Ziska and Caulfield 2000). Therefore, global warming is a public health concern because it has the potential to alter the timing and abundance of aeroallergens (Beggs 2004), which could result in increased symptoms in those with allergic rhinitis or asthma.

An underappreciated but important consideration is the interactive effects of CO_2_ with other known or predicted changes in climate and their impact on biotic systems. For example, CO_2_ may be driving the warming that results in earlier springs, but plants will experience both effects at the same time (i.e., higher CO_2_ and a longer growing season). Hence, it is important to study how climate variables will interact to drive plant responses.

In this study, we sought to increase our understanding of the potential response of common ragweed (*Ambrosia artemisiifolia* L.), a late-season flowering allergenic plant, to springtime climate variability and examine interactive effects of increased CO_2_. We performed a controlled environment study with simulated changes in the timing of spring, at both ambient and future predicted CO_2_ levels, to test whether variability in the onset of spring alters the rate and magnitude of ragweed development, flowering phenology, and seasonal pollen production and whether atmospheric CO_2_ concentrations directly alter rag-weed development and productivity and influence plant responses to climatic variability.

## Materials and Methods

Common ragweed (*A. artemisiifolia*) is a C_3_ plant (a plant that uses a 3 carbon compound for CO_2_ fixation during photosynthesis which should thrive in enriched CO_2_ atmospheres) common to roadsides and disturbed habitats throughout most of the United States and Canada (Bassett and Crompton 1975). It is monoecious, with separate male and female flowers borne on the same plant on distinct axillary branches, allowing for independent control of allocation to sexes (Payne 1963).

Seeds of *A. artemisiifolia* collected from wild populations in Woodstock, Illinois, were vernalized by sowing seeds in six growth containers containing compost (Pro-Mix, Red Hill, PA) and storing in a refrigerator at 4°C until ready for germination. Two trays at a time were transferred from cold conditions to the glasshouses at three 15-day intervals, creating three temporal cohorts that would simulate variability in the onset of the growing season and would include anticipated advances of spring several decades into the future. One tray from each cohort was placed in 380 ppm (ambient) and the other at 700 ppm (elevated) CO_2_ concentration. From each pair of trays, seedlings were chosen that all germinated on the same day; the germination dates (23 May 2002, 7 June 2002, and 22 June 2002) were also at 15-day intervals. The middle cohort approximates the germination date of plants in the Boston area (Rogers C, personal observation).

Approximately 15 days after their germination, we transplanted 24 seedlings from each tray into 6-dry-quart–capacity growth containers (22.23 cm diameter × 21.59 cm deep). Soil in each container was composed of a 4:1 mix of Pro-Mix compost and washed sand (Quickrete Co., Atlanta, GA). The soil mixture was amended with slow-release 14:14:14 nitrogen:phosphorous:potassium fertilizer (Osmocote; Scott’s, Marysville, OH), and plants were watered daily.

The glasshouses consist of six modules structured as three blocks, each block having two modules of differing CO_2_ concentrations (380 and 700 ppm). Containers were arranged in the modules according to their CO_2_ and temporal cohort (i.e., eight plants in each of three temporal cohorts, in each of three glasshouse modules, at both low and high CO_2_, for a total of 144 plants). Day/night temperatures were maintained at 26/21°C. Ambient glasshouse light levels were approximately 70% of full sun, supplemented with 6 hr of light daily (1000 to 1600 hr) from overhead metal halide lamps, thus allowing plants to experience natural variation in day length. Temperature, CO_2_, and light were computer-controlled for all modules, and we used corn plants (*Zea mays*) to help maintain a constant CO_2_ concentration in the low-CO_2_ modules. In each module, temporal cohorts of ragweed plants were separated, and the positions of the containers within each treatment were randomized at intervals to minimize edge effects. Cohorts were grown at a foliar density of approximately nine plants per square meter. We recorded measurements of flower phenology and date of first pollen release for each ragweed plant throughout the experiment.

We chose five male floral spikes at random from each plant in the first two cohorts and three from each plant in the third cohort at each CO_2_ level and placed a 5 cm × 25 cm polyethylene bag over each selected spike, similar to the procedure described in Ziska and Caulfield (2000). On one side of the bag near the bottom, we cut a small slit and placed the spike inside. The slit was then taped shut and the bag left to collect pollen shed by the spike, with the tops of the bags left open for ventilation. After pollen production had stopped, we measured the length of the bagged flower spikes, cut each at the base, and stored the spike in the collection bag at −20° C until ready for evaluation. Bags in which water accumulated due to watering or heavy condensation were discarded, leaving 477 individual inflorescences.

After senescence, we harvested plants over 3 days from 16 through 18 September. Plant height and number of inflorescences were recorded, the plants were cut at the base, and all flower spikes were removed and placed in bags separate from the vegetative material. We measured the length of each floral spike on each plant. Roots were washed clean of dirt and also placed in separate bags. All plant material was dried at 70°C for 48 hr, and we recorded separate dry weight measurements for all roots, flowers, and vegetative material.

For each bagged flower spike, pollen was recovered by twice repeated 30-sec vortexing in a wash solution (distilled water with 0.02% Tween 20) in 15 mL Falcon tubes, followed by 5-min centrifugation (2,500 rpm; relative centrifugal force = 600). Pollen recoveries from the spike and pollen rinsed from the polyethylene bag were combined in a total volume of 2.0 mL wash solution. We determined the number of pollen grains per spike by calculating the pollen concentration in the wash suspension from microscopic counts using a glass hemacytometer (Hausser Scientific, Horsham, PA).

For each inflorescence, we estimated pollen production *p_ij_* from an allometric model based on log inflorescence length, time of dormancy release, CO_2_ concentration, total number of inflorescences, total weight of inflorescences, and days to anthesis:


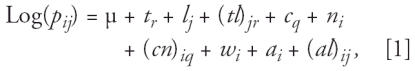


where μ is a constant and *j* indexes each inflorescence of log length *l_j_* on plant *i* with number of inflorescences *n_i_*, total inflorescence weight *w_i_*, and days to anthesis *a_i_*, dormancy release at time *t_r_*, and grown under CO_2_ concentration *c_q_*. Additional interaction terms did not improve model prediction. We estimated whole-plant pollen production, *p_i_*, as the sum of pollen production over all inflorescences on each plant.

We used a two-way factorial design with time of dormancy release crossed with CO_2_ treatment and CO_2_ nested within glasshouse wing to assess the responses to the timing of dormancy release and CO_2_, and we modeled estimated pollen count, inflorescence number, inflorescence weight, aboveground biomass, plant height, days to anthesis, and date of anthesis. Time was included as a fixed term. Glasshouse wing and CO_2_ within wing were included as random terms to permit broad inference. We included the time × CO_2_ interaction as a fixed term because plants were individually randomized.

## Results

The impact of variability in the onset of spring under scenarios of ambient and elevated CO_2_ was assessed through several bio-mass (plant height, aboveground biomass), phenological (days to anthesis, anthesis date), and reproductive measures (number of inflorescences, inflorescence length, total weight of inflorescences, pollen production).

The model estimating whole-plant pollen production explained 62% of the variation in measured pollen counts from 477 inflorescences collected from 141 of 144 individual plants. Pollen production per inflorescence was most strongly associated with inflorescence length, number of inflorescences per plant, and days to anthesis (Table 1). The negative association between inflorescence number and pollen production suggests a tradeoff, with some plants producing fewer pollen-rich inflorescences and others producing more inflorescences each producing less pollen per unit length.

We examined the interaction of time of dormancy release and CO_2_ concentration, and the results are presented in Table 2. Significant time × CO_2_ interaction terms were found in each of estimated pollen count, inflorescence number, inflorescence weight, aboveground biomass, and plant height (marginally). CO_2_ treatment did not significantly affect days to anthesis or anthesis date.

**Figure 1:**
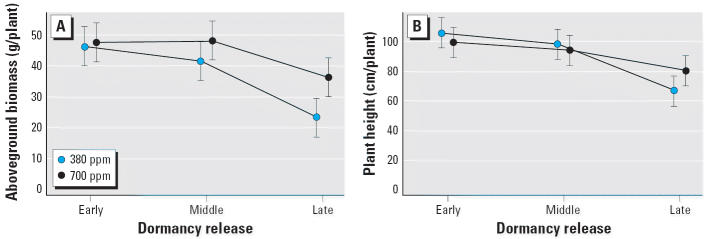
End-of-season biomass measures of *A. artemisiifolia*: aboveground biomass (*A*) and plant height (*B*) for three springtime dormancy release cohorts at two CO_2_ concentrations (380 ppm and 700 ppm). Error bars indicate 95% confidence intervals.

We calculated least-square means for each level of time and CO_2_ along with 95% confidence limits for the measures of biomass, reproductive effort, phenology, and pollen productivity. Plants in early spring cohorts had significantly greater aboveground biomass and height than did the late cohort, as shown in Figure 1, with the greatest difference between early and late cohorts. Little additional gain in biomass or height was achieved between middle and early cohorts under either CO_2_ condition, perhaps indicating that near-maximal growth occurred in the longer growing seasons. However, in cohorts released from dormancy later, plants grown in elevated CO_2_ acquired significantly greater height and weight. Hence, elevated CO_2_ appears to have a greater impact on increasing biomass when plants are younger and/or smaller.

**Figure 2:**
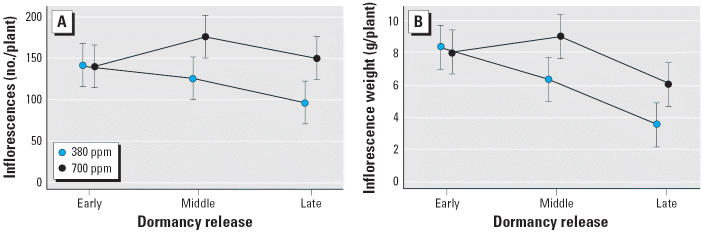
End-of-season reproductive measures of *A. artemisiifolia*: number of inflorescences (*A*) and inflorescence weight (*B*) for three springtime dormancy release cohorts at two CO_2_ concentrations (380 ppm and 700 ppm). Error bars indicate 95% confidence intervals.

Earlier release from dormancy also increased reproductive effort measured by the number of inflorescences and inflorescence weight (Figure 2). There was a continuous trend toward a greater number of, and heavier, inflorescences in the middle and early cohorts at ambient CO_2_ levels. In the early cohort, there was no difference in reproductive effort for plants grown at ambient versus high CO_2_. Interestingly, at high CO_2_, plants from the middle cohort had the highest number and heaviest inflorescences. At high CO_2_, inflorescences were significantly heavier and more abundant in both the middle and later cohorts than at ambient CO_2_ levels.

**Figure 3:**
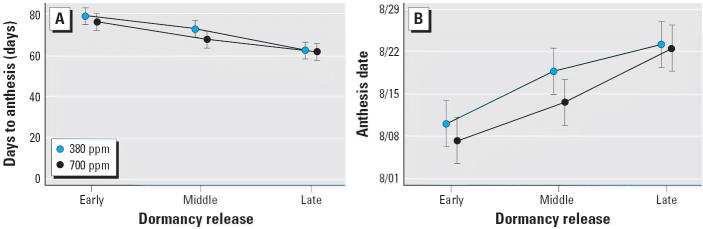
Phenology measures of *A. artemisiifolia*: number of days to anthesis (*A*) and anthesis date (*B*) for three springtime dormancy release cohorts at two CO_2_ concentrations (380 ppm and 700 ppm). Error bars indicate 95% confidence intervals.

We also examined the influence of growing season length and CO_2_ on phenological responses of days to anthesis and anthesis date (Figure 3). Photoperiodic control of flower initiation is well documented in *A. artemisiifolia* and is similar to many other late-summer–flowering plants. Therefore, little difference in the number of days to flowering (anthesis) and anthesis date was expected. Logically, plants released from dormancy earlier had a longer time until flowering. However, surprisingly, the anthesis date (date on which first pollen release was recorded) differed among the three cohorts. There was a consistent trend toward a later date of first anthesis in the later cohorts. Therefore, although flower initiation is reportedly under photoperiodic control (Lewis et al. 1991), anthesis apparently is not. There was no effect of CO_2_ on the number of days until anthesis or anthesis date for any cohort.

**Figure 4:**
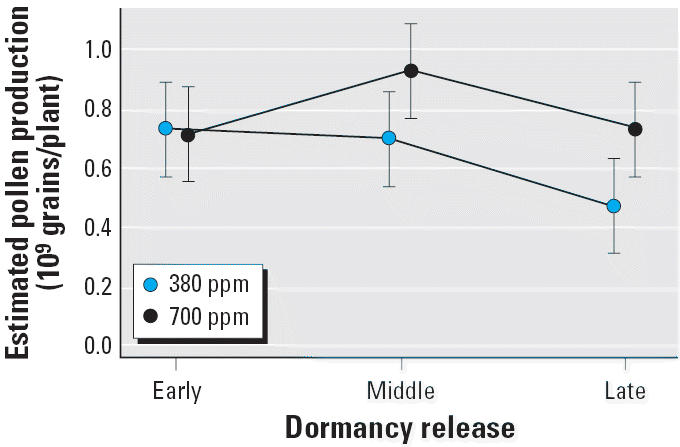
Pollen production in *A. artemisiifolia* for three springtime dormancy release cohorts grown at two CO_2_ concentrations (380 ppm and 700 ppm). Error bars indicate 95% confidence intervals.

Finally, at ambient CO_2_ levels, estimates of whole-plant pollen production, based on parameters outlined in the model above, were higher in earlier cohorts (Figure 4). At ambient CO_2_ levels, the simulated early spring cohort produced 54.8% more pollen compared with plants released from dormancy late. High CO_2_ did not further increase pollen production relative to ambient CO_2_ in the early cohort, but increased pollen production was observed in the middle (32.0% increase, *p* = 0.0506) and late (55.0% increase, *p* = 0.0240) cohorts.

## Discussion

This study is the first to assess the potential impact of earlier arrival of spring, and the interaction with CO_2_, as expected with global warming and increased climate variability, on pollen productivity in allergenic plants. Based on the current rate of phenological advances (5 days/decade) (Root et al. 2003), the degree of advancement used in this study is similar to what might be expected three to six decades in the future. Our simulated effect of earlier spring dormancy release allowed ragweed plants to accumulate more resources through the season, thereby increasing biomass and reproductive effort. Plants in ambient CO_2_ released from dormancy earlier had increased height and weight, more and heavier inflorescences, and 54.8% higher pollen production compared with those released 30 days later. Increased temperatures, which would accompany earlier spring and elevated CO_2_ under future climate regimes, although not studied in these experiments, might also affect pollen production.

Because increasing atmospheric CO_2_ is assured for the next several decades, and it is unknown how CO_2_ might interact with climatic variables to influence plant responses, we also determined the additional interactive effects of elevated CO_2_ with variations in the onset of spring. We found that there was no additional advantage to plants in the earliest cohort grown under high CO_2_. However, the number and weight of inflorescences were significantly greater at high CO_2_ relative to ambient levels for plants in both the middle and late cohorts. Increased biomass and pollen production was also significantly higher in the late cohort at high CO_2_ levels. Hence, the reproductive disadvantage of a shorter growing season could be ameliorated when plants are grown in elevated CO_2_.

It is a well-known phenomenon of chamber studies that the advantage of elevated CO_2_ is greatest early in plant development but diminishes over time (Drake et al. 1997). In essence, plants exhibit acclimation to elevated CO_2_ with age (Long et al. 2004) or perhaps as a result of resource depletion due to the confines of growth within pots (Drake et al. 1997). We found the least difference in productivity between plants in ambient and elevated CO_2_ for the earliest cohort. Early-cohort plants in elevated CO_2_ may have had an early advantage but then acclimated over time, and/or the longer growing season may have been sufficient for ambient-CO_2_ plants to make up the early difference. In contrast, in the latest cohort with the shortest growing season—and hence the least amount of time for ambient-CO_2_ plants to make up the early difference in productivity—elevated-CO_2_ plants had significantly greater biomass, number and weight of inflorescences, and pollen production relative to ambient-CO_2_ plants. These results highlight the importance of examining the interactive effects of CO_2_ with other climate variables in order to understand the implications of climate change.

The climate variability that stems from global warming is a significant concern. Our results show that variability in the onset of spring elicits a strong increase in pollen production in early seasons at ambient CO_2_ concentrations. However, in elevated CO_2_, although pollen productivity is enhanced, it is less sensitive to variability in season onset. Hence, in future climates with elevated CO_2_, we predict pollen production will be just as robust in years with late springs as in years with early springs. Overall, pollen production in ragweed can be expected to increase significantly under predicted future climate conditions.

Our results are consistent with the findings of other greenhouse and chamber studies on ragweed that have shown a 60–90% increase in pollen productivity with elevated CO_2_ (700 or 600 ppm) compared with current ambient levels (Wayne et al. 2002; Ziska and Caulfield 2000). Of course, the ability to generalize results of closed environment experiments to natural field populations is an important issue; however, *A. artemisiifolia* also appears to be a strong competitor in mixed populations in elevated CO_2_ (Bazzaz and Garbutt 1988). In addition, similar results on pollen productivity have been found in field studies. In cities, because of proximity to industrial and vehicular sources, atmospheric CO_2_ concentrations and temperatures are much higher than in the surrounding rural areas (Idso et al. 2002). Using a naturally occurring gradient in Baltimore, where temperature and CO_2_ are elevated by 1.8–2.0°C and 30% (to ~500 ppm), respectively, compared with outlying areas, Ziska et al. (2003) found that in experimental ragweed plots, plants increased biomass and pollen production with the degree of urbanization. This brings to light two facts: Plants in the field are responding similarly to effects modeled in glasshouse experiments, and urban plants are currently experiencing changed atmospheric conditions that are altering their pollen productivity now, not decades into the future.

Both allergies and asthma have been increasing worldwide in recent decades, significantly above that expected from better diagnosing or increased reporting (von Mutius 1998). Although the trend may be showing early signs of leveling off (Hertzen and Haahtela 2005; Lawson and Senthilselvan 2005), there still is a much greater proportion of the population that is vulnerable to allergen exposure than ever before. Ragweed pollen allergens are some of the most potent in North America, and roughly 10% of the population is sensitized (Gergen et al. 1987). Diesel particles from truck and vehicle exhaust have been shown to act synergistically with pollen allergens to exacerbate disease (Hauser et al. 2003) and are now thought to be an important factor in the recent rise in allergic disease (Riedl and Diaz-Sanchez 2005). Hence, an important question is whether greater ragweed pollen production (with or without diesel particle coexposure) will lead to an increase in the frequency or severity of asthma and allergy symptoms, or to new sensitizations and a further increase in development of allergic disease.

## Conclusion

The effects of global warming are complex, but studies of their impact on biotic communities clearly point toward secondary effects that could be detrimental to human health. Our study of *A. artemisiifolia* under conditions that simulate future levels of atmospheric CO_2_ and increased temperatures shows that one effect—increased production of allergenic pollen—could strongly affect the significant proportion of the population with pollen allergies as climate change progresses. Because the results of this study suggest that, under future conditions of global warming and elevated CO_2_, pollen seasons will be more intense and could start earlier than expected, pollen forecasting and pollen avoidance strategies for sensitized individuals will be particularly important. Finally, we emphasize the importance of studying interactions between multiple predicted climate change parameters, in this case, the interactive effects of elevated CO_2_ and variability in the onset of spring. Our study suggests that under future predicted greenhouse gas emissions and associated climate conditions, either an early spring onset or variability in spring onset along with elevated CO_2_, there will be an overall increase in ragweed pollen production.

## Figures and Tables

**Table 1:** Regression coefficients for the model estimating whole-plant pollen productivity of *A. artemisiifolia*.

Parameter	Symbol	Group	Coefficient	SE	*t*-Value	*p*-Value
Intercept	μ		3.47	1.63	2.13	0.0340
Log inflorescence length	*l_j_*		5.48	0.652	8.40	< 0.0001
Time of dormancy release	*t_r_*	Early	0.00	—	—	—
		Middle	1.63	0.370	4.41	< 0.0001
		Late	2.37	0.505	4.68	< 0.0001
Log inflorescence length × time of release	(*tl*)*_jr_*	Early	0.00	—	—	—
		Middle	−0.665	0.151	−4.40	< 0.0001
		Late	−0.837	0.198	−4.24	< 0.0001
No. of inflorescences	*n_i_*		−0.00503	0.000851	−5.91	< 0.0001
CO_2_ concentration	*c_q_*	380 ppm	0.00	—	—	—
		700 ppm	−0.448	0.153	−2.93	0.0036
No. of inflorescences × CO_2_	(*cn*)*_iq_*	380 ppm	0.00	—	—	—
		700 ppm	0.00297	0.00106	2.79	0.0055
Weight of inflorescences	*w_i_*		0.0509	0.0157	3.24	0.0013
Days to anthesis	*a_i_*		0.119	0.0207	5.73	< 0.0001
Log inflorescence length × days to anthesis	(*al*)*_ij_*		−0.0518	0.00840	−6.17	< 0.0001

**Table 2:** Effects of time of release, CO_2_ concentration, and the interaction of time and CO_2_ modeled on measures of biomass, reproduction, phenology, and pollen production.

Response	Term	*F*-value^a^	*p*-Value
Pollen count (estimated)	Time	8.49	0.0003
	CO_2_	2.54	0.2519
	Time × CO_2_	4.39	0.0143
Inflorescence number	Time	2.91	0.0579
	CO_2_	13.12	0.0685
	Time × CO_2_	3.58	0.0306
Inflorescence weight	Time	40.24	< 0.0001
	CO_2_	3.61	0.1979
	Time × CO_2_	8.66	0.0003
Aboveground biomass	Time	42.78	< 0.0001
	CO_2_	5.06	0.1534
	Time × CO_2_	4.13	0.0181
Plant height	Time	23.80	< 0.0001
	CO_2_	0.07	0.8125
	Time × CO_2_	2.97	0.0546
Days to anthesis	Time	62.40	< 0.0001
	CO_2_	1.63	0.3299
	Time × CO_2_	1.25	0.2890
Anthesis date	Time	49.42	< 0.0001
	CO_2_	1.63	0.3299
	Time × CO_2_	1.25	0.2890

a For the *F*-statistic, numerator degrees of freedom: time = 2, CO_2_ = 1, time × CO_2_ = 2; denominator degrees of freedom: time = 134, CO_2_ = 2, time × CO_2_ = 134 (except for plant height, where denominator degrees of freedom for time and time × CO_2_ are 133).
